# Molecular analysis of the *PAX6* gene for congenital aniridia in the Korean population: Identification of four novel mutations

**Published:** 2012-02-19

**Authors:** Shin Hae Park, Man Soo Kim, Hyojin Chae, Yonggoo Kim, Myungshin Kim

**Affiliations:** 1 Seoul St. Mary’s Hospital, Department of Ophthalmology and Visual Science, College of Medicine, The Catholic University of Korea, Seoul, Korea; 2 Department of Laboratory Medicine, College of Medicine, The Catholic University of Korea, Seoul, Korea

## Abstract

**Purpose:**

To analyze the paired box gene 6 (*PAX6*) in Korean patients with congenital aniridia.

**Methods:**

Genomic DNA was isolated from peripheral blood leukocytes of 22 aniridia patients in 18 unrelated families. Polymerase chain reaction was performed for all 14 exons of *PAX6* followed by bidirectional sequencing.

**Results:**

Fourteen different kinds of mutations were detected in 16 of 18 unrelated families (mutation detection rate: 88.9%), including four novel mutations; c.658G>T (p.Glu220*), c.464delG (p.Ser155Thrfs*52), c.87_90dupTGTA (p.Glu31Cysfs*26), and c.642A>C (p.Arg214Ser), among which the former three mutations induce premature termination of PAX6 protein translation. Approximately 92.9% of identified mutations lead to the premature termination of the protein resulting from 7 nonsense mutations (50.0%), 3 splicing errors (21.4%), 2 deletions (14.3%), and 1 insertion (7.1%).

**Conclusions:**

Most of the mutations identified in Korean aniridia patients lead to the premature truncation of the PAX6 protein, supporting that PAX6 protein haploinsufficiency causes the classic aniridia phenotype. We also found four novel *PAX6* mutations associated with aniridia.

## Introduction

Congenital aniridia (OMIM 106210) is a rare ocular malformation that affects the development of multiple ocular structures and is caused by a mutation in the paired box gene 6 (*PAX6*) located on chromosome 11p13 [1-3]. Iris hypoplasia is the most obvious sign, but a broad spectrum of disorders can manifest [2-4]. Many patients have corneal opacities, cataracts, nystagmus, and foveal and optic nerve hypoplasia. Aniridia typically causes severe visual impairment; the major causative factor of this condition is foveal hypoplasia [5].

The incidence of congenital aniridia ranges from 1:64,000 to 1:96,000 [5]. In two-thirds of the cases, it is inherited in an autosomal dominant fashion with almost complete penetrance and variable expressivity; and the remaining one-third of the cases are sporadic [1,6,7]. Some sporadic cases have a risk of developing Wilms tumor as a part of WAGR (Wilms tumor, aniridia, genitourinary abnormalities, and mental retardation; OMIM 194072), which is caused by deletion of both *PAX6* and Wilms’ tumor gene (*WT1*) in the 11p13 region.

*PAX6* was isolated as a candidate gene for aniridia by positional cloning in 1991 [8]. Heterozygous mutations are found in about 40%–80% of all non-syndromic aniridia patients [1,9-11]. Numerous *PAX6* mutations have been detected in aniridia patients (Online Human PAX6 Allelic Database), and premature termination of the PAX6 protein is the most frequent type of mutation [11].

Although about 60 cases of congenital aniridia have been reported in Korea since the first report in 1977, little is known about the molecular characterization of congenital aniridia in Koreans [12-15]. Here, we analyzed *PAX6* in 22 Korean aniridic patients and identified the genetic aberrations and genotype-phenotype correlations.

## Methods

This study was approved by the Ethics Committee of Seoul St. Mary’s Hospital, The Catholic University of Korea (KC11RISI0722). Informed consent was obtained from the patients.

We evaluated 22 patients in 18 unrelated aniridia families in Seoul St.Mary’s Hospital. The age, gender, visual acuity, family history, and previous ocular history of the patients were recorded. Thorough ocular examinations were performed, including best-corrected visual acuity (BCVA), intraocular pressure (IOP), and refractive measurement and slit lamp biomicroscopy of the anterior segment and fundus. After receiving informed consent, blood samples were collected from all patients for DNA extraction and *PAX6* analysis.

Genomic DNA was isolated from peripheral blood leukocytes with the QIAmp DNA Mini Kit (Qiagen, Hamburg, Germany). The DNA was quantified spectrophotometrically using a ND-1000 (Nanodrop Technologies Inc., Wilmington, DE). All 14 exons (including an alternatively spliced exon 5a) of *PAX6* were amplified using the primers as previously described (Table 1) [10]. For all amplicons, the genomic DNA was denatured at 95 °C for 5 min followed by 35 cycles of denaturation at 95 °C for 30 s, annealing at 60 °C for 30 s, extension at 72 °C for 1 min, and final extension at 72 for 5 min. The PCR products were examined by agarose gel (1.5%) electrophoresis followed by staining the gel in ethidium bromide (0.5 μg/ml), which then was visualized under ultraviolet (UV) light in a gel documentation system (Gel Doc 1000; Biorad, Hercules, CA). PCR amplicons were bidirectionally sequenced with the Big Dye terminator v3.1 cycle sequencing kit (Applied Biosystems, Foster City, CA) using the ABI PRISM 3100 Genetic Analyzer (Applied Biosystems). RefSeq ID: NM_000280.3 was used for cDNA nucleotide numbering.

**Table 1:** List of primers used to perform amplification and sequencing of the 14 *PAX6* exons.

***PAX6* gene**	**Primer sequences for *PAX6* (5′→3′)**	**Tm (°C)**
Exon 1	F-CTCATTTCCCGCTCTGGTTC	56
	R-AAGAGTGTGGGTGAGGAAGT	
Exon 2	F-TTATCTCTCACTCTCCAGCC	56
	R-AAGCGAGAAGAAAGAAGCGG	
Exon 3	F-TCAGAGAGCCCATCGACGTAT	56
	R-CTGTTTGTGGGTTTTGAGCC	
Exon 4	F-TTGGGAGTTCAGGCCTACCT	56
	R-GAAGTCCCAGAAAGACCAGA	
Exon 5	F-CCTCTTCACTCTGCTCTCTT	56
	R-ATGAAGAGAGGGCGTTGAGA	
Exon 5a	F-TGAAAGTATCATCATATTTGTAG	50
	R-GGGAAGTGGACAGAAAACCA	
Exon 6	F-GTGGTTTTCTGTCCACTTCC	56
	R-AGGAGAGAGCATTGGGCTTA	
Exon 7	F-CAGGAGACACTACCATTTGG	56
	R-ATGCACATATGGAGAGCTGC	
Exon 8	F-GGGAATGTTTTGGTGAGGCT	56
	R-CAAAGGGCCCTGGCTAAATT	
Exon 9	F-GTAGTTCTGGCACAATATGG	54
	R-GTACTCTGTACAAGCACCTC	
Exon 10	F-GTAGACACAGTGCTAACCTG	56
	R-CCCGGAGCAAACAGGTTTAA	
Exon 11	F-TTAAACCTGTTTGCTCCGGG	56
	R-TTATGCAGGCCACCACCAGC	
Exon 12	F-GCTGTGTGATGTGTTCCTCA	56
	R-TGCAGCCTGCAGAAACAGTG	
Exon 13	F-CATGTCTGTTTCTCAAAGGGA	54
	R-GAACAATTAACTTTTGCTGGCC	

## Results

### Ocular phenotypes

Table 2 shows the ocular phenotypes of 22 patients in 18 unrelated families tested. The male/female ratio was 0.59. The percentage of sporadic cases was 36.4%. The mean age of the patients was 19.4±14.7 years. Total aniridia was demonstrated in 20 patients and partial aniridia in 2 patients (patients 6-1 and 17). Glaucoma, cataracts, keratopathy above grade II, and foveal hypoplasia were also observed in addition to iris aplasia in the patients, as detailed below. Nephroblastoma did not develop during the follow-up period.

**Table 2:** Ocular findings in Korean patients with congenital aniridia.

**Case No**	**Age/gender**	**Inheritance**	**BCVA (OD/OS)**	**Nystagmus**	**Keratopathy**	**Cataract**	**Glaucoma**	**Macular hypoplasia**	**Comments**
1-1	27/F	Familial	0.1/0.1	+	Grade II	+	-	+	
1-2	3Mon/F	Familial	F&F (+)	+	Grade IV	-	-	uncheckable	Corneal opacity
2	34/M	Sporadic	0.2/0.16	+	Grade I	+	-	-	
3	15/M	Sporadic	0.25/0.16	+	-	+	-	-	
4	1/F	Sporadic	F&F (+)	+	Grade II	+	-	+	
5	24/M	Familial	0.04/0.04	+	Grade II	+	+, eyedrops	+	
6-1	31/F	Sporadic	HM/0.02	+	Grade IV	+	+, eyedrops	+	Partial aniridia
6-2	1/M	Familial	F&F (+)	+	-	+	-	+	
7-1	8/F	Familial	0.1/FC 30 cm	+	Grade III	+	-	+	
7-2	40/M	Familial	FC 30 cm /0.1	+	Grade IV	+	+, eyedrops	+	
8-1	48/F	Familial	0.02/0.02	+	Grade IV	+	-	+	
8-2	15/F	Familial	0.16/0.06	+	Grade I	+	+, surgery	+	Valve implant
9	21/M	Sporadic	0.32/0.2	+	-	-	-	+	
10	3/M	Sporadic	FC10 cm/LP-	+	Grade IV	+	+, eyedrops	uncheckable	Corneal opacity
11	30/M	Familial	0.1/FC 30 cm	+	Grade IV	+	-	+	
12	15/F	Familial	FC50 cm/0.1	+	Grade I	+	-	+	
13	8/M	Sporadic	0.16/0.2	+	Grade II	+	-	+	
14	4/M	Familial	0.16/0.16	+	-	-	+, eyedrops	+	
15	30/M	Familial	0.02/0.1	+	Grade III	+	+, eyedrops	+	
16	48/F	Familial	0.04/0.04	+	Grade IV	+	-	+	
17	16/M	Familial	0.06/0.06	+	Grade I	+	-	+	Partial aniridia
18	8Mon/M	Sporadic	F&F (+)	+	-	-	-	-	

M: Male; F: Female; F&F: Fix and follow; PD: paired domain; LNK: linker domain; HD: Homeodomain; PST: proline-, serine-, and threonine-rich transregulatory domain. Keratopathy was graded as follows: grade 0, clear; grade 1, peripheral mudding with ingrowth of neovascular tissue not exceeding 1 mm from the limbal arch; grade II, peripheral neovascularization in at least the peripheral half of the cornea, corneal clouding, and subepithelial fibrosis; grade III, involvement of the central cornea.

Glaucoma was observed in 7 of 22 patients (31.8%). Six patients could maintain IOP within the normal range with topical anti-glaucoma medications, but one female (patient 12) required surgical treatment with Ahmed valve implantation in her left eye at 12 years old.

Cataracts were seen in 18 of 22 patients (81.8%), and 6 had received cataract surgery. Congenital corneal opacity was observed in two children (patients 2 and 14), who required penetrating keratoplasty. A fundus examination was performed in 20 patients who did not have severe corneal or lens opacity. Foveal hypoplasia, defined as the absence of a foveal reflex, was found in 17 patients (85%).

### Genetic analysis of *PAX6*

The patients’ molecular findings are summarized in Table 3. Fourteen different mutations were detected in 16 of 18 unrelated families (88.9%). We found four novel mutations, including c.87_90dupTGTA, c.464delG, c.642A>C, and c.658G>T, in addition to 10 known mutations: c.11–2A>G, c.19G>T, c.301delG, c.317T>A, c.524–2A>G, c.607C>T (n=3), c.718C>T, c.901C>T, c.949C>T (n=2), and c.1183+2T>C [9,16-26].

**Table 3:** Molecular Findings in Korean patients with congenital aniridia.

**Case No**	**Mutation**	**Exon/Intron**	**Domain**	**mRNA/protein effect**
1-1	c.11-2A>G	Intron 4	PD	Splicing error
1-2	c.11-2A>G	Intron 4	PD	Splicing error
2	c.19G>T	Exon 5	PD	p.Gly7*
3	c.87_90dup TGTA†	Exon 5	PD	p.Glu31Cysfs*26
4	c.301delG	Exon 6	PD	p.Glu101Lysfs*23
5	c.317T>A	Exon 6	PD	p.L106*
6-1	c.464delG†	Exon 7	LNK	p.Ser155Thrfs*52
6-2	c.464delG†	Exon 7	LNK	p.Ser155Thrfs*52
7-1	c.524-2A>G	Intron 7	LNK	Splicing error
7-2	c.524-2A>G	Intron 7	LNK	Splicing error
8-1	c.607C>T	Exon 8	LNK	p.Arg203*
8-2	c.607C>T	Exon 8	LNK	p.Arg203*
9	c.607C>T	Exon 8	LNK	p.Arg203*
10	c.642A>C†	Exon 8	HD	p.Arg214Ser
11	c.658G>T†	Exon 8	HD	p.Glu220*
12	c.718C>T	Exon 9	HD	p.Arg240*
13	c.901C>T	exon10	PST	p.Gln310*
14	c.949C>T	Exon 11	PST	p.Arg317*
15	c.949C>T	Exon 11	PST	p.Arg317*
16	c.1183+2T>C	Intron 12	PST	Splicing error
17	Not detected			
18	Not detected			

A mark with † indicates the four novel mutations identified in this study.

The types of mutations were as follows: 11 single nucleotide substitutions, including 1 missense mutation (7.1%), 7 nonsense mutations (50.0%), and 3 intronic mutations that lead to splicing errors (21.4%), in addition to 3 indel mutations that resulted in frameshifts (21.4%).

The DNA-binding domains (DBDs) of the PAX6 protein were composed of the 128 amino acid paired domain (PD) and the 61 amino acid homeodomain (HD) separated by a linker region (LNK). The proline-, serine-, and threonine-rich transregulatory (PST) domain in the COOH-terminal region was composed of 152 amino acids. In this study, 5 kinds of mutations (35.7%) occurred in the PD, and 3 kinds of mutations (21.4%) occurred in the LNK, HD, and PST domains. Relatively fewer mutations have been detected in the PST domain considering its long size.

**Figure 1:**
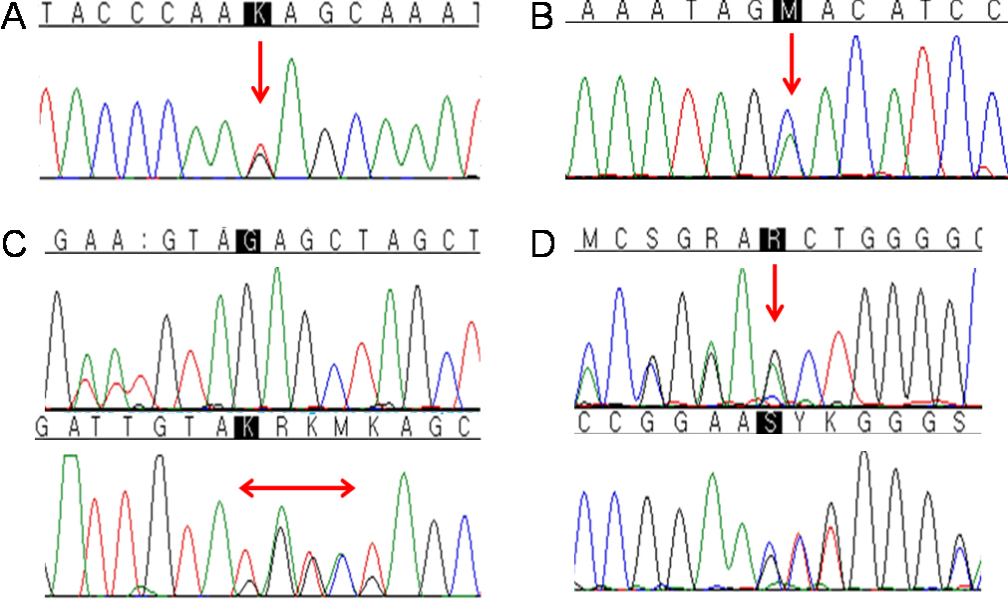
Four novel *PAX6* mutations were identified in this study. **A**-**D**: Sequencing chromatograms of c.658G>T, c.642A>C, c.87_90dupTGTA, and c.464delG, respectively.

Four novel mutations were detected in this study (Table 3, Figure 1). Patient 3 showed the c.87_90dupTGTA (p.Glu31Cysfs*26) mutation in exon 5 within the PD, which resulted in a premature termination due to the frame-shift. Patients 6–1 (mother) and 6–2 (son) possessed a 1-bp deletion, c.464delG (p.Ser155Thrfs*52), in exon 7, which causes a frameshift and premature termination of translation in the LNK domain of the PAX6 protein. Patient 10 was 3 year-old male and had a novel missense mutation, c.642A>C (p.Arg214Ser), in exon 8 within the HD. This mutation was predicted to be not tolerable by SIFT analysis and possibly damaging by PolyPhen analysis. This child had a severe clinical manifestation of aniridia with marked corneal opacity and increased IOP in both eyes. This mutation was not detected in his unaffected mother. Patient 11 showed a novel nonsense mutation, c.658G>T (p.Glu220*), in exon 8, which results in premature termination within the LNK domain.

Single nucleotide variation (SNV) c.766–12C>T (rs667773) was detected in 3 patients in 2 probands (7–1, 7–2, 10) who represented c.524–2A>G and c.642A>C, respectively.

### Genotype-phenotype correlation

Ophthalmic and genetic findings exhibited inter- and intrafamilial phenotypic variabilities of the disease. Patient 1–2 had bilateral congenital corneal opacities presumed to be accompanying Peters anomaly, which were not observed in her mother (Patient 1–1) with the same splicing error mutation (c.11–2A>G). In the family with the c.464delG mutation (Patients 6–1 and 6–2), one showed complete aniridia, and the other showed partial aniridia. Patient 8–2 had juvenile onset glaucoma in both eyes, which was not detected in Patients 8–1 and 9 with the same nonsense mutation (c.607C>T).

We did not find any phenotypic differences according to the location of the identified genotype. Nineteen patients had total aniridia irrespective of the domain of identified mutations, except for Patient 6–1 with the LNK domain frameshift mutation. The ocular phenotypes in patients with three truncating mutations in the PST domains, which retained the intact DNA-binding domains, were comparable to that with mutations within the PD and HD domains.

## Discussion

In this report, we described *PAX6* mutations in 22 Korean aniridia patients from 18 unrelated families. The mutation detection rate was 88.9% (16/18). The mutation spectrum of *PAX6* in aniridia was highly biased, as 92.9% of identified mutations included 7 nonsense mutations (50.0%), 3 splicing errors (21.4%), 2 deletions (14.3%), and 1 insertion (7.1%), leading to the premature truncation of the protein and one missense mutation inducing an amino acid change in the HD domain. Interestingly, 4 novel mutations were identified in this study, including 3 mutations (c.87_90dupTGTA, c.464delG, and c.658G>T) leading to the premature termination of the PAX6 protein and one missense mutation (c.642A>C).

The phenotype of aniridia could be explained by the haploinsufficiency of the PAX6 protein, in which the mutated PAX6 protein does not have any transcriptional activity and the remaining single normal copy of *PAX6* is not enough to produce a sufficient threshold level of biologically active PAX6 protein to initiate the transcription of its target genes [11,27,28]. A critical dose of PAX6 protein is required to initiate the transcription of its downstream target genes for normal eye development [28]. Nonsense-mediated decay, which is the process in which mRNAs containing premature termination codons are degraded before they produce large amounts of truncated proteins, is relevant to the pathomechanism of aniridia because the major mutations detected in aniridia are truncations. Haploinsufficiency in the mutants in the COOH-terminal half of the PAX6 protein could be explained by dominant-negative effects, which could be caused by competition for DNA-binding between truncated PAX6 proteins and wild-type PAX6 proteins. Some truncated mutants have 3–5 fold higher affinities to various DNA binding sites when compared with the wild-type PAX6 [27].

The clinical manifestations associated with aniridia express variable phenotypes. We did not find any phenotypic differences according to the location of the identified genotypes. Our results also exhibited interfamilial (Patients 8–2 and 9) and intrafamilial (Patients 1–1 and 1–2, Patients 6–1 and 6–2, and Patients 8–1 and 8–2) phenotypic variabilities of the disease. Atchaneeyasakul et al. reported that the total aniridia phenotype was associated with mutations at the COOH-terminus, whereas partial aniridia patients carried mutations that resulted in a loss of the homeodomain with or without a loss of the paired domain [29]. However, our results are not consistent with that finding. Total aniridia was observed in 19 patients irrespective of the domain of identified mutations, except for patient 6–1 with the frameshift mutation within the LNK. The reason for variable phenotypes among individuals with the same mutation is unclear. The variable expressivity could be explained by subtle differences in PAX6 protein levels and the ratio of mutant to wild type PAX6 protein or the interactions with other factors [27]. Mutations behind the HD could potentially lead to more severe phenotypes than truncating mutations within the DNA-binding domains, such as the PD and HD [11,28]. Generally, the *PAX6* missense mutation occurs less frequently in aniridia and has a tendency to be associated with milder phenotypes [18,30]. A PAX6 protein with an amino acid substitution could still retain some residual activity and result in partial haploinsufficiency. Some missense mutations might have the potential to impair the proper folding of the PAX6 protein and compromise the normal three dimensional structure. In our 3-year old male (Patient 10) carrying the novel missense mutation c.642A>C (p.Arg214Ser), other factors than the mutated PAX6 protein might contribute to his severe phenotype.

Accodring to the *PAX6* mutation database, the most frequent *PAX6* mutations in aniridia are c.607C>T, c.718C>T, c.949C>T, and c.1267dupT (Online Human PAX6 Allele Database) [9,11]. The former three muations were also identified in this study. The distribution of the identified mutations was as follows: 35.7% in the PD, 21.4% in the LNK, 21.4% in the HD, and 21.4% in the PST domains. Definite mutational hot spots were not observed in our study. In two patients, a *PAX6* mutation was not identified with direct DNA sequencing throughout the whole gene. Exon deletions and deletions of control regions can be the cause of isolated aniridia, so that tests used to identify gene copy number, such as quantitative PCR, mutiplex ligation-dependent probe amplification (MLPA), and array comparative genomic hybridization, may be helpful to clarify such cases.

c.766-12C>T (rs667773) was found in 2 probands. This SNV has been considered as probably a neutral polymorphism and defined as variation in 1000 Genomes. Various mutations were detected with this SNV including c.949C>T, c.277G>A, c.607C>T, c.1267dupT in the PAX6 mutation database (Online Human PAX6 Allelic Database), and we cannot find any cosegregation pattern.

Generally, it is recommended to perform several analyses in anridia to obtain the maximum detection yield, as aniridia could be caused by different type of genetic aberrations. Chromosomal rearrangement and deletion can be detected by karyotype analysis espcially in the cases of WAGR or the aniridia patients presenting other malformation [24]. Fluorescence in situ hybridization and MLPA can detect cryptic deletion of *PAX6* effectively [24,31]. Detection of *PAX6* mutations was performed using direct sequencing method combined with or without mutation detection screening tools such as DHPLC (denaturing high performance liquid chromatography) or SSCP (single-strand conformation polymorphism). Mutation detection rate by direct sequencing of *PAX6* was variable as follows; 47% (18/38) in Chinese [32], 49% (34/70) in Caucasian [31], 30% (9/30) in Mexican [9], and 67% (4/6) in Thai patients [29]. To our best knowledge, our *PAX6* mutation detection rate of 88.9% is the one of highest rates by single test alone. One of the estimated reason of our high mutation detection rate is the characteristics of patients included in this study. Most of patients had clinically definite non-syndromic aniridia with total absence of iris (20/22). The other possible reason to improve detection rate is that we performed bidirectional sequencing in all samples because the mutations in aniridia patients were distributed throughout the whole exon and intron of *PAX6*. Based on our result, the bidirectional DNA sequencing including whole exon and intron-exon boundary of *PAX6* could be recommended as the first screening test for the molecular confirmation of aniridia, especially when it is not combined with other systemic abnomalities such as renal tumor, genitourinary abnormalities, and mental retardation.

In conclusion, most of the mutations identified in Korean aniridia patients lead to the premature truncation of the PAX6 protein, supporting that haploinsufficiency of the PAX6 protein causes the classic aniridia phenotype. Also, we found four novel *PAX6* mutations associated with aniridia.
